# Boosting effect of IL-7 in interferon gamma release assays to diagnose *Mycobacterium tuberculosis* infection

**DOI:** 10.1371/journal.pone.0202525

**Published:** 2018-08-29

**Authors:** Hellen Hiza, Lukas Fenner, Jerry Hella, Davis Kuchaka, Mohamed Sasamalo, Thomas Blauenfeldt, Gibson Kibiki, Reginald A Kavishe, Francis Mhimbira, Morten Ruhwald

**Affiliations:** 1 Ifakara Health Institute, Bagamoyo, Tanzania; 2 Swiss Tropical and Public Health Institute, Basel, Switzerland; 3 University of Basel, Basel, Switzerland; 4 Institute of Social and Preventive Medicine, University of Bern, Bern, Switzerland; 5 Kilimanjaro Clinical Research Institute, Kilimanjaro, Tanzania; 6 Statens Serum Institut, Center for Vaccine Research, Copenhagen, Denmark; 7 East African Health Research Commission, Bujumbura, Burundi; 8 Kilimanjaro Christian Medical University College, Tumaini University, Kilimanjaro, Tanzania; Universita degli Studi di Palermo, ITALY

## Abstract

**Background:**

A quarter of the world’s population is estimated to be infected with *Myobacterium tuberculosis (Mtb)*. Infection is detected by immune response to *M*. *tuberculosis* antigens using either tuberculin skin test (TST) and interferon gamma release (IGRA’s), tests which have low sensitivity in immunocompromised. IL-7 is an important cytokine for T-cell function with potential to augment cytokine release in in-vitro assays. This study aimed to determine whether the addition of IL-7 in interferon-gamma release assays (IGRAs) improves its diagnostic performance of *Mtb* infection.

**Methods:**

44 cases with confirmed TB and 45 household contacts without TB were recruited and 1ml of blood was stimulated in two separate IGRA’s tube set: one set of standard Quantiferon TB gold tubes mitogen, TB antigen and TB Nil; one set of customized Quantiferon TB gold tubes with added IL-7. Following IFN-γ and IP-10 release was determined using ELISA.

**Results:**

We found that the addition of IL-7 led to significantly higher release of IFN-γ in individuals with active TB from 4.2IU/ml (IQR 1.4–6.9IU/ml) to 5.1IU/ml (IQR 1.5–8.1IU/ml, p = 0.0057), and we found an indication of a lower release of both IFN-γ and IP-10 in participants with negative tests.

**Conclusions:**

In TB cases addition of IL-7 in IGRA tubes augments IFN-γ but not IP-10 release, and seems to lower the response in controls. Whether IL-7 boosted IGRA holds potential over standard IGRA needs to be confirmed in larger studies in high and low TB incidence countries.

## Introduction

The need for improved diagnostics and ultimately a new vaccine for tuberculosis (TB) cannot be overemphasized. An estimated quarter of the global population is infected with *Mycobacterium tuberculosis* (Mtb) [1], and in 2015 alone, 10.4 million people developed TB and 1.8 million people died of TB [2]. The new WHO end TB strategy aims to reduce new TB cases by 90% until 2035 through better use of available technology along with introduction of better diagnostic tools and better vaccines [3]. The implementation plan behind these ambitious goals introduced a shift from active case finding to a test-to-treat strategy of individuals infected with Mtb at risk of developing TB.

A major roadblock in the test-to-treat strategy is the poor understanding of Mtb infection and absence of strong correlates for development of active TB disease in those infected. Current state-of-art consider Mtb infection as a dynamic condition wherein the outcome of the infection (progression or control) is strongly dependent on the initial encounter between immune system and bacterium and risk factors for progression e.g., HIV infection [4,5]. The adaptive immune response plays a central role in the control of the infection especially through induction of CD4^+^ T cells [6]. Several small and highly recognized antigens secreted by Mtb are essential factors for the virulence of the bacterium [7]. These antigens includes ESAT-6 and its secretion partner CFP-10 and the fact that these antigens are recognized by the majority of infected individuals has enabled the development of the interferon gamma release assays (IGRA), an in-vitro diagnostic concept for specific detection of infection [8,9]. In IGRAs, immune competent cells either in the form of whole blood (as in the Quantiferon Gold In-Tube (QFT)) or purified peripheral mononuclear cells (PBMCs, as in the T-SPOT.TB test) are stimulated in-vitro with ESAT-6 and CFP-10 and the degree of immune recognition is determined by measurement of the antigen specific release of interferon-gamma (IFN-γ) [9]. A major limitation to the use of IGRAs is that the tests rely on live cells to generate the result. This require a robust laboratory and sample handling framework as well as technical expertise for efficient sample handling and analysis, which can be a challenge in most settings wherein the test-to-treat strategy is intended to be implemented [9]. Another challenge with the IGRAs is their poor predictive value for development of active TB [10] and IGRAs can be false negative in high risk groups e.g., HIV infected [9].

Attempts to improve the robustness of the IGRA technology have been ongoing for a decade, and it is now recognized that IFN-γ is only one of several markers with immunodiagnostic potential, among whom IP-10 is a leading alternative expressed in 100 fold higher levels [11]. Attempts to stabilize and prolong the responsiveness of the immune competent cells in the IGRA includes blockade of anti-inflammatory cytokines (e.g. IL-10) [12], elimination of granulocytes during cell preparation for the T-SPOT.TB [13], as well as addition of immune mediators which role it is to improve survival of T cells [14]. IL-7 is a key regulator of CD4^+^ T cells survival, both long term and cells responding to antigen. Previous studies have shown that IL-7 increases both frequency and spot size in the T-SPOT.TB assay improving overall diagnostic performance of the IGRA [12,15,16]. The aim of this study was to develop a ready-to-use vacutainer tube system comprising IL-7 and the QFT Ag cocktail, and to assess its performance in terms of magnitude of cytokine release and diagnostic performance.

## Material and methods

### Study setting, study population, and procedures

The study was conducted within the frame of an ongoing prospective cohort from November 2014 to April 2016 study of adult (≥18 years) TB patients and household contact (controls) in the Temeke district, Dar es Salaam, Tanzania (TB-DAR) [17] Cases were defined as TB patients with a positive sputum smear result for acid-fast bacilli [AFB] and a positive TB culture on solid media. Household contact controls were exposed to a TB case in the household, but with a negative sputum smear microscopy and Xpert MTB/RIF results, matched to the case by age (±5 years) and whenever possible by sex. We did not include a control group with individuals unlikely to have latent tuberculous infection (LTBI) because the study setting was in the high TB endemic area.

Study participants were routinely screened for malaria, helminth infections (Kato-Katz, Baermann, urine filtration) at the time of enrolment. We recruited 50 cases and 50 controls for this study. After exclusion of study participants with missing samples or not enough sample volume (<1ml), we finally included 44 cases and 45 controls.

#### IL-7 Tube preparation

Under sterile conditions, caps were removed from QFT nil and antigen tube (Qiagen, DE) and recombinant IL-7 (Thermo Fisher Scientific, Waltham, MA, USA) diluted in sterile water to a final concentration of 1μg/ml was added in a 2μl droplet to the side of the tube[18]. Tubes were covered with sterile tin foil, frozen at -80°C for 24h before freeze drying. Following, tubes were recapped and kept in plastic zipper bags with desiccant and stored for 4°C until use. On day of use, vacuum sufficient for 1ml blood draw was created using a syringe and needle as described elsewhere (19).

#### Interferon-gamma release and IP-10 release testing

Whole blood was drawn into vacutainer tubes and within 4 hours placed in a 37°C incubator for 18 24 hours. Here after plasma was isolated by centrifugation and samples were stored at -20°C for later analysis. IFN-γ levels were detected using sandwich ELISA as per manufacturers’ instructions (Qiagen, Germany) and IP-10 was measured in plasma diluted x30 using a qualified in-house ELISA described previously (20)

#### Statistical analysis

The antigen specific release of IFN-γ and IP-10 was determined after subtraction of the unstimulated (nil) sample from the antigen sample. IFN-γ and IP-10 release was compared between groups using non-parametric test (Kruskal-Wallis test). Diagnostic potential independent of cut off was assessed by comparing cases and controls using Receiver Operations Characteristics Curve analysis (ROC). After application of cut off (0.35IU/ml) the diagnostic potential was assessed using McNemars test. Analyses were performed in SAS 9.2 and GraphPad Prizm 7.02.

### Ethics statement

The study protocol was approved by the institutional review board of the Ifakara Health Institute (IHI; reference no. IHI/IRB/No 04–2015) and the Medical Research Coordinating Committee of the National Institute of Medical Research (NIMR; reference no. NIMR/HQ/R.8c/Vol.I/357) in Tanzania, and the ethics committee of north-west and central Switzerland (EKNZ; reference no: UBE-15/42). Written informed consent was obtained from all study participants.

## Results

### Study population

We analyzed 44 TB cases and 45 household controls without TB. TB cases were more likely male (68.1% vs. 28.9%), had a lower median body mass index (BMI) at the time of TB diagnosis (17.6 vs. 25.2 kg/m2), and were more likely to be HIV-positive (27.3% vs. 4.4%) compared to controls, whereas distributions of age, sex, smoking, and intestinal infection were similar. Among household contacts, 48.9% had positive QFT status shown and one test indeterminate (Table 1).

**Table 1:** Patient characteristics of tuberculosis cases and household contact controls without TB, Dar es Salaam, Tanzania.

Characteristic	All	Cases	Controls
**Total, n (%)**	**89 (100)**	**44 (49.4)**	**45 (50.6)**
**Age**, years, median (IQR)	32 (25.6–42.0)	33.92 (28–43.3)	30.44 (23.4–38.6)
**Female sex**, n (%)	50 (56.2)	18 (40.9)	32 (71.1)
**BMI**, kg/m2, median (IQR)	21.05 (17.8–25.24)	17.58 (15.98–20.4)	25.22 (21.98–32.1)
**Current smoker**, n (%)	12 (13.48)	6 (13.64)	4 (13.33)
**Occupation**, n (%)			
Unemployed	32 (36.0)	11 (25.0)	21 (46.7)
Employed	57 (64.0)	33 (75.0)	24 (53.3)
**Monthly income (USD)**, n (%)			
<100	42 (47.2)	23 (52.3)	19 (42.2)
≥100	47 (52.8)	21 (47.7)	26 (57.8)
**Education level**, n (%)			
No/primary	77 (86.5)	39 (88.6)	38 (84.4)
Secondary/University	12 (13.5)	5 (11.4)	7 (15.6)
**Symptoms** ^1^, n (%)			
Coughing	64 (71.9)	42 (95.5)	22 (48.9)
Fever	43 (48.3)	32 (72.7)	11 (24.4)
Night sweats	54 (60.7)	37 (84.1)	17 (37.8)
Unexplained weight loss	47 (52.8)	42 (95.5)	5 (11.1)
**HIV infection**, n (%)	14 (15.7)	12 (27.3)	2 (4.4)
CD4^+^ count (cells/μl), median (IQR)^2^		140 (87.5–170)	-
**Full blood counts** ^1^ (10^9^ cells/L)			
Neutrophils, median (IQR)	4.1 (2.8–5.7)	5.0 (3.8–6.4)	2.7 (2.1–3.3)
Platelets, median (IQR)	278.5 (207.0–376.5)	350 (247.0–420.0)	244.0 (196.0–299.0)
Red blood cells, mean (±SD)	4.75 (0.8)	4.54 (0.8)	4.9 (0.7)
**Hemoglobin** (g/dL) ^1^, mean (±SD)	11.88 (2.2)	11.27 (2.4)	12.44 (1.8)
**Any helminth infection** ^1^, n (%)	20 (22.5)	11 (25.0)	9 (20.0)
**TB patient category**			
New case	43 (97.7)	43 (97.7)	-
Relapse	1 (2.3)	1 (2.3)	-
**AFB smear microscopy result**, n (%)			
Scanty	2 (4.55)	2 (4.55)	-
1+	13 (29.6)	13 (29.6)	-
2+	20 (45.5)	20 (45.5)	-
3+	9 (20.5)	9 (20.5)	-
**QFT Status**			
Positive	60 (68.2)	38 (86.4)	22(48.9)
Negative	28 (31.8)	6(13.6)	22(48.9)
Indeterminate	-	-	1(2.2)

AFB, Acid fast bacilli; BMI, body mass index; IQR, Interquartile range; SD, Standard deviation; TB, Tuberculosis; USD, United States Dollar

^1^ at the time of TB diagnosis (cases) or enrolment

^2 ^CD4^+^ cell counts were only available among HIV positive TB cases (all were naïve to antiretroviral therapy)

### Impact of IL-7 on IFN-γ and IP-10 release in cases and controls

**Fig 1:**
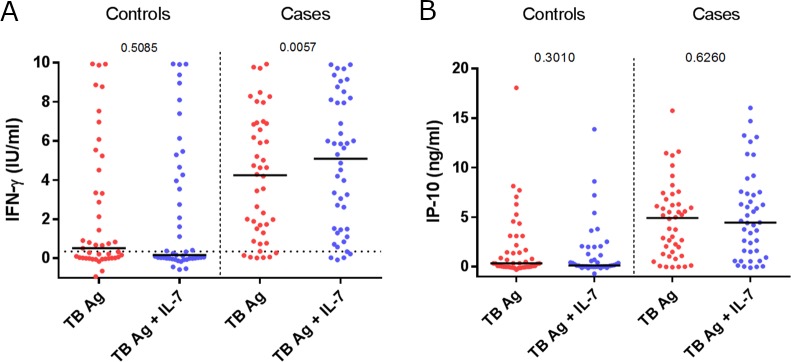
**A comparison of the effect of added IL-7 on antigen specific release of IFN-γ (A) and IP-10 (B) in TB cases (n = 44) and endemic controls (n = 45).** Whole blood was incubated 18–24 hours whereupon plasma was isolated for later analysis with ELISA.

The antigen specific release of IFN-γ was higher in TB patients median 4.2IU/ml (IQR 1.4–6.9IU/ml) compared to controls median 0.5IU/ml (IQR 0.0–3.9IU/ml, p<0.0001) (Fig 1). Adding IL-7 led to a more categorical separation between cases and controls, through a significantly higher antigen specific release in cases from median 4.2IU/ml (IQR 1.4–6.9IU/ml) to 5.1IU/ml (IQR 1.5–8.1IU/ml, p = 0.0057) and an overall albeit not significantly lower release in controls from median 0.5IU/ml (IQR 0.0–3.9IU/ml) to 0.2IU/ml (IQR 0.0–4.7). Interestingly, IFN-γ levels in the nil tube were unaffected by the presence of IL-7 (p = 0.501, data not shown).

Antigen specific release of IP-10 was significantly higher in cases compared to controls, median (4.9ng/ml (IQR 1.3–6.9ng/ml) vs 0.3ng/ml (IQR 0.0–2.6ng/ml, p<0.0001) (Fig 1). Of note, we did not find a significantly higher IP-10 release with IL-7 in stimulated samples from cases as seen with IFN-γ (Fig 1B), but IL-7 did seem to drive a similar non-significant contraction in the IP-10 release in controls as seen for IFN-γ (0.3ng/ml (IQR 0.0–2.6ng/ml) to 0.1ng/ml (IQR 0.0–1.6ng/ml) (Fig 1).

**Fig 2:**
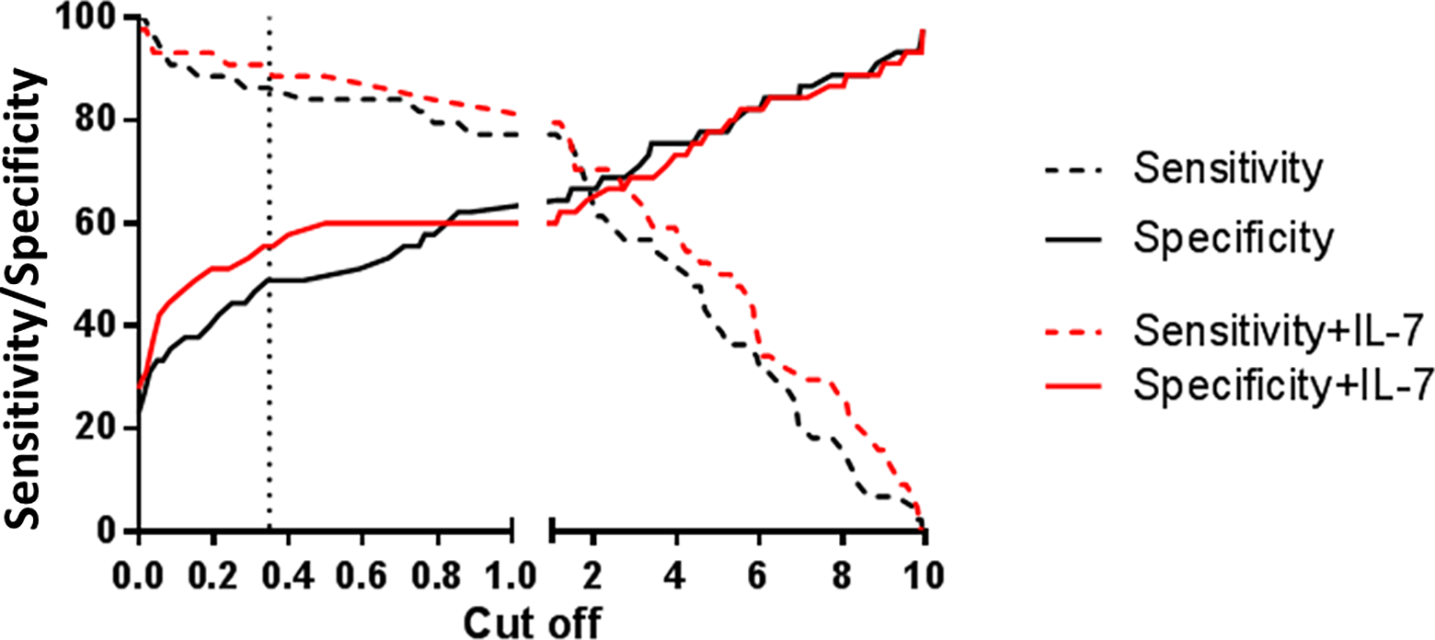
Comparison of added IL-7 for positive test on sensitivity and specificity in IFN-γ release assay. The dotted line indicates the 0.35 IU/ml cut off recommended by the manufacturer.

The ROC curve analysis did not show a significant impact of added IL-7 on either IFN- γ or IP-10 (area under the curve IFN- γ with IL-7 0.73 [95% CI 0-63-0.84], IFN-γ without IL-7: 0.71 [95% CI 0.60–0.82] and IP-10: 0.76 (95% CI 0.66–0.86, data not shown). However, by plotting sensitivity and specificity with or without IL-7 against potential cut offs, it was illustrated how the contracted IFN-γ response and herewith lower variability seen in the IL-7 treated control samples, has potential for improved diagnostic sensitivity in tests with cut offs <0.8IU/ml (Fig 2).

### Diagnostic performance of IL-7 coated tubes

**Fig 3:**
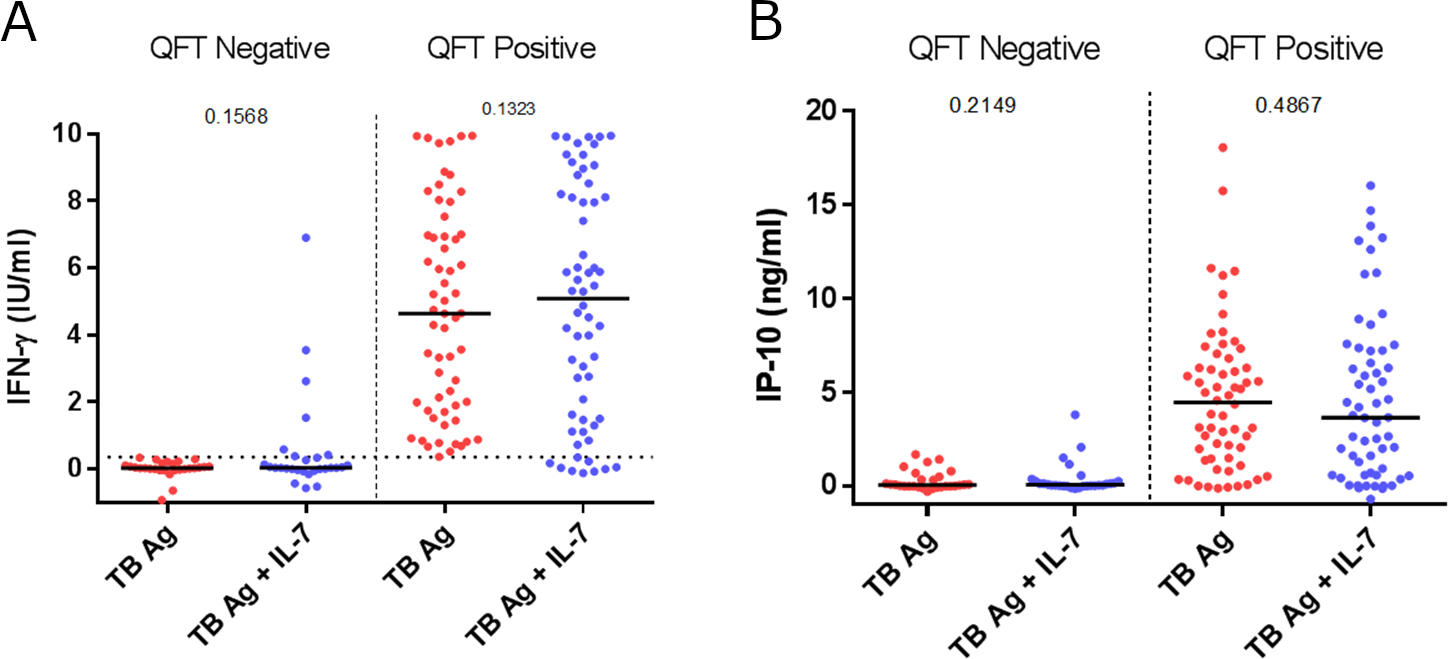
**A comparison of the effect of added IL-7 on antigen specific release of IFN-γ (A) and IP-10 (B) in Quantiferon (QFT) negative (n = 28) and QFT positive (n = 60).** Whole blood was incubated 18–24 hours whereupon plasma was isolated for later analysis with ELISA. Lines denote median, p-value calculated using Kruskal-wallis test.

Splitting study participants by QFT status demonstrated that the presence of IL-7 did not lead to significant differences in the magnitude of IFN-γ or IP-10 release within the groups (Fig 3). After applying the manufacturers recommended cut off of 0.35IU/ml for IFN-γ and 0.75 ng/ml IP-10 tests, there were no significant change the number of positive results in either cases (n = 38 (86%) and n = 39 (88%)) or household contacts controls (n = 22 (49%) and n = 20 (44%), S1 Table). IP-10 had comparable diagnostic performance with QFT in cases; n = 37 (84%) and controls; n = 18 (40%) (S2 Table).

### Discordant results

Addition of IL-7 resulted in discordance in 7/44 (16%) cases and 10/45 (22%) controls in the IFN-γ based tests (Table 2). Two reversions (one case and one control) and two conversions (one cases and one control) were caused by smaller differences in IFN-γ release (<0.50IU/ml), changes more likely caused by analytical variability than an IL-7 effect [19]. However, the remaining 13 participants had discordant results, which could not be explained by analytical variability. Nine QFT positive revert to negative with added IL-7 during the stimulation. Five were controls and except for one who reverted from 0.66IU/ml to 0.17IU/ml the remaining five controls were “true reverters” with dramatic reduction in release >2.87IU/ml. Only one was positive with IP-10 without IL-7 and all were IP-10 negative when IL-7 was added (Table 2). These study participants with discordant responses tended to have a lower BMI, but were all HIV-negative and otherwise comparable to the remaining cohort.

**Table 2:** Test results, IFN-γ and IP-10 release levels in participants with discordant outcome of IFN-γ based test.

	Status	QFT result	QFT+IL-7 result	IP-10 result	IFN-γ (Ag-Nil)	IFN-γ+IL7 (Ag-Nil)	IP-10 (Ag-Nil)	Dif. +/- IL-7
**Conversion**	Case	NEGATIVE	POSITIVE	NEGATIVE	0.04	6.9	-0.01	6.86
	Case	NEGATIVE	POSITIVE	NEGATIVE	0.28	0.58	0.13	0.30*
	Case	NEGATIVE	POSITIVE	NEGATIVE	0.06	1.53	-0.05	1.47
	Case	NEGATIVE	POSITIVE	POSITIVE	0.15	2.62	1.04	2.47
	Control	NEGATIVE	POSITIVE	NEGATIVE	-0.01	3.54	0.07	3.55
	Control	NEGATIVE	POSITIVE	NEGATIVE	-1.72	0.38	0.03	2.10
	Control	NEGATIVE	POSITIVE	NEGATIVE	0.76	6.14	0.35	5.38
** **	Control	NEGATIVE	POSITIVE	NEGATIVE	0.33	0.42	0.68	0.09*
**Reversion**	Case	POSITIVE	NEGATIVE	NEGATIVE	0.74	-0.08	0.51	-0.82
	Case	POSITIVE	NEGATIVE	POSITIVE	0.77	0.34	2.89	-0.43*
	Case	POSITIVE	NEGATIVE	POSITIVE	4.64	0.22	3.75	-4.42
	Control	POSITIVE	NEGATIVE	POSITIVE	5.54	0.05	7.05	-5.49
	Control	POSITIVE	NEGATIVE	POSITIVE	3.32	0.03	8.15	-3.29
	Control	POSITIVE	NEGATIVE	NEGATIVE	0.66	0.17	-0.12	-0.49*
	Control	POSITIVE	NEGATIVE	NEGATIVE	2.87	-0.01	0.01	-2.88
	Control	POSITIVE	NEGATIVE	NEGATIVE	7.53	-0.06	-0.07	-7.59
	Control	POSITIVE	NEGATIVE	NEGATIVE	6.08	-0.12	0.33	-6.20

* denotes participants with a difference in IFN-γ release between IL-7 boosted and standard QFT within the range of expected analytical variability of the assay.

## Discussion

In this study, we explored added value of IL-7 on whole blood stimulation in IFN-γ and IP-10 release assays. We found that IL-7 led to significantly higher release of IFN-γ in individuals with active TB p = 0.0057 and we found an indication of a lower release of both IFN-γ and IP-10 in controls. The impact of IL-7 on IFN-γ release was most pronounced in controls with a positive QFT.

IL-7 is an essential cytokine for T cell development as well as for the survival and homeostasis of mature T cells [20–22]. Disruption of the IL-7 axis leads to severe lymphopenia, and administration of IL-7 in HIV seropositive people and patients suffering from other types of lymphopenia, results in rapid replenishment of the T cell pool, promote T cell expansion and normalization of lymphocyte functions [21,23]. These interesting effects of IL-7 are being explored as adjunct therapy e.g., after bone marrow transplantation [21,24,25]. The IL-7 receptor is almost exclusively expressed on lymphoid cells, and IL-7 receptor ligation activates several pathways including Jak-Stat5 (to promote T cell differentiation), the PI3K/Akt pathway (responsible for survival), as well as upregulation of several anti-apoptotic genes including Bcl-2 and MCL1 [20,21,24].

The biological effects of IL-7 suggest it has benefits to promote cytokine release in cell mediated immune response assays like the IGRA. When primed with specific antigens, IL-7 has an effect on TCR activation in mature T cell through expansion of T cells specific to the antigens [22] in concert with stabilized INF-γ signaling and augmented IL-2 receptor expression also on terminally differentiated T cells [20,21,26].

These findings have been translated to the IGRA test concept, where IL-7 primed with Mtb specific antigens has been shown to increase IFN-γ mRNA levels and increase IFN-γ protein production (2–14 fold increase) [16,18,27] and promote larger spot size in the T-SPOT.TB IGRA [15,28]. Our study is the first confirming the effects of higher Mtb antigen specific IFN-γ release in a larger material and our use of a field friendly method of IL-7 prepared blood collection tubes opens for further larger studies (Fig 2). Our findings also expands previous knowledge by demonstrating a more categorical separation between TB cases and controls potentially due to stabilization of IFN-γ translation as reported in previous studies [18,27,28].

In agreement with other reports, addition of IL-7 did not augment the antigen specific IP-10 release in cases, but it had an effect by stabilizing the unspecific release in nil samples and in the controls [18]. This absence of augmented release is a somewhat surprising finding as IP-10 release by the antigen presenting cell (APC) is augmented by IFN-γ, TNF and other cytokines released by the specific T cell recognizing its antigen on the APC [11]. A likely explanation to this apparent paradox could be that the IL-7 mediated effects on IFN-γ release occur at a late stage of the relatively short 18–24 hour stimulation assay, whereas the co-stimulatory effects of IFN-γ on STAT1 activation and IP-10 release is more effective if it occurs early during the incubation [11]. Kinetic studies are required to elucidate the interplay of these potential effects, however the IL-7 effect of lower variability in IP-10 release among controls, supports this hypothesis.

One of the limitations in the IGRAs especially in the high endemic setting, is the frequent occurrence of low level responses in the ‘uncertainty zone’ between 0.2–0.7IU/ml, responses with high likelihood of conversion and reversion with serial testing [19,29,30]. QFT positives with IFN-γ release falling within the ‘uncertainty zone’ (0.35 to 0.7IU/ml) are particularly likely to revert, and it was very recently shown that these individuals had the same low risk of progression to TB as the QFT negatives[30]. Moreover, the group of strict converters (defined as a change from <0.2 to >0.7IU/ml) comprised almost all individuals who later developed TB disease, suggesting that interpretation of serial QFT tests taking the ‘uncertainty zone’ into account could improve preventive treatment by only offering treatment to strict converters[30]. A major contributor to low-level responses in the ‘uncertainty zone’ is the biological variability of IFN-γ release in this rather complex in-vitro assay. Our findings suggest that some of the biological variability of the QFT can be curbed by IL-7 mediated stabilization of the IFN-γ response, allowing for a clearer separation between cases and endemic controls [18,27,28]. Further studies are needed to assess whether IL-7 has a potential to improve management of preventive treatment.

Although our exploratory study was insufficiently powered to demonstrate a significance in diagnostic sensitivity, it suggested that in the presence of IL-7 there was also a tendency for reversion of positives to negative in controls. Overall the IGRA positive population have an increased risk of developing TB, however studies suggest this risk is relatively low[31], further studies are required to determine if the observed discordance with added IL-7 leads to a more accurate risk stratification.

Limitation to be taken into account was missing data for the few patients which were taken out of analysis reducing the sample size. The small size of this study limits the generalizability of the findings but is acceptable for proof of concept assessment of novel concepts like freeze-drying IL-7 in the QFT tubes. The use of endemic controls in exploratory studies is another limitation preventing true assessment of the specificity of the IL-7 concept, however with no indication of altered specificity through increased unspecific IFN-γ secretion in this report and in the literature, warrants our approach[15,18,28].

In conclusion, we found that the addition of IL-7 in IGRA tubes has a positive effect on IFN-γ expression, but not on IP-10 expression. This suggested a potential superior diagnostic potential for IL-7 boosted IGRAs. Another potential clinical implication suggested by the data is improved diagnostic sensitivity in high-risk groups. Prompted by the observed lower IFN-γ release in the endemic control group suggests that the cut off can be lowered for an IL-7 augmented IGRA to enable improved diagnostic sensitivity in children and immunosuppressed individuals such as e.g., HIV infected. The diagnostic potential of IL-7 needs to be confirmed in larger studies in high and low TB incidence countries, however our demonstration of a IL-7 coated ready to use antigen tube should make such evaluation feasible

## Supporting information

S1 Table Quantiferon vs., IL-7 boosted Quantiferon calculated with the standard 0.35 IU/ml cut off. (DOCX)

S2 Table Quantiferon vs., IP-10 determined in Quantiferon supernatant, IP-10 cut off at 0.75 ng/ml. (DOCX)
