# Supplementary material for: Boosting effect of IL-7 in interferon gamma release assays to diagnose Mycobacterium tuberculosis infection
Source: PLoS One. 2018 Aug 29;13(8):e0202525. doi: 10.1371/journal.pone.0202525 (PMC6114790; doi:10.1371/journal.pone.0202525)
Supplement: S1 Table — (DOCX) [file pone.0202525.s001.docx]

S1 Table. Quantiferon vs., IL-7 boosted Quantiferon calculated with the standard 0.35 IU/ml cut off.

|  | **Cases (QFT + IL-7)** | | | |  | **Control (QFT + IL-7)** | | | | | | | |
| --- | --- | --- | --- | --- | --- | --- | --- | --- | --- | --- | --- | --- | --- |
|  |  | **+** | **-** | Total |  | |  | | **+** | | **-** | | Total |
| **QFT, n (%)** | **+** | 35 (89.7) | 3 (60.0) | 38 (86.4) |  | **+** | | 16 (80.0) | | 6 (24.0) | | 22 (48.9) | |
|  | **-** | 4 (10.3) | 2 (40.0) | 6 (13.4) |  | **-** | | 4 (20.0) | | 19 (76.0) | | 23 (51.1) | |
|  | Total | 39 (88.6) | 5 (11.4) | 44 (100.0) |  | Total | | 20 (44.4) | | 25 (55.6) | | 45 (100.0) | |

Note; n, number, **+**, positive; **-**, negative; QFT, Quantiferon; IL-7, Interleukin 7
