# Supplementary material for: Boosting effect of IL-7 in interferon gamma release assays to diagnose Mycobacterium tuberculosis infection
Source: PLoS One. 2018 Aug 29;13(8):e0202525. doi: 10.1371/journal.pone.0202525 (PMC6114790; doi:10.1371/journal.pone.0202525)
Supplement: S2 Table — (DOCX) [file pone.0202525.s002.docx]

S2 Table. Quantiferon vs., IP-10 determined in Quantiferon supernatant, IP-10 cut off at 0.75 ng/ml

|  | **Cases (IP-10)** | | | |  | **Control (IP-10)** | | | |
| --- | --- | --- | --- | --- | --- | --- | --- | --- | --- |
|  |  | **+** | **-** | Total |  |  | **+** | **-** | Total |
| **QFT, n (%)** | **+** | 35 (94.6) | 3 (42.9) | 38 (86.4) |  | **+** | 15 (83.3) | 7 (25.9) | 22 (48.9) |
|  | **-** | 2 (5.4) | 4 (57.1) | 6 (13.6) |  | **-** | 3 (16.7) | 20 (74.1) | 23 (51.1) |
|  | **Total** | 37 (84.1) | 7 (15.9) | 44 (100.0) |  | Total | 18 (40.0) | 27 (60.0) | 45 (100.0) |

Note; n, number, **+**, positive; **-**, negative; QFT, Quantiferon; IP-10, Interferon gamma induced protein - 10
